# Impaired tumor growth and angiogenesis in mice heterozygous for *Vegfr2 (Flk1)*

**DOI:** 10.1038/s41598-018-33037-2

**Published:** 2018-10-03

**Authors:** Sunday S. Oladipupo, Ashraf Ul Kabir, Craig Smith, Kyunghee Choi, David M. Ornitz

**Affiliations:** 1 Department of Developmental Biology, Washington University School of Medicine, St. Louis, MO 63110 USA; 2 Department of Pathology and Immunology, Washington University School of Medicine, St. Louis, MO 63110 USA; 3 Present Address: Bio-TDR, Lilly Research Laboratories, Eli Lilly and Company, Indianapolis, IN 46285 USA; 4 Graduate School of Biotechnology, Kyung Hee University, Yong In, South Korea

## Abstract

VEGF signaling through its tyrosine kinase receptor, VEGFR2 (FLK1), is critical for tumor angiogenesis. Previous studies have identified a critical gene dosage effect of *VegfA* in embryonic development and vessel homeostasis, neovascularization, and tumor growth, and potent inhibitors of VEGFR2 have been used to treat a variety of cancers. Inhibition of FGFR signaling has also been considered as an antiangiogenic approach to treat a variety of cancers. Inhibition of VEGFR2 with neutralizing antibodies or with pharmacological inhibitors of the VEGFR tyrosine kinase domain has at least short-term efficacy with some cancers; however, also affects vessel homeostasis, leading to adverse complications. We investigate gene dosage effects of *Vegfr2*, *Fgfr1*, and *Fgfr2* in three independent mouse models of tumorigenesis: two-stage skin chemical carcinogenesis, and sub-cutaneous transplantation of B16F0 melanoma and Lewis Lung Carcinoma (LLC). Mice heterozygous for *Vegfr2* display profound defects in supporting tumor growth and angiogenesis. Unexpectedly, additional deletion of endothelial *Fgfr1* and *Fgfr2* in *Vegfr2* heterozygous mice shows similar tumor growth and angiogenesis as the *Vegfr2* heterozygous mice. Notably, hematopoietic deletion of two alleles of *Vegfr2* had minimal impact on tumor growth, with little effect on angiogenesis, reinforcing the importance of endothelial *Vegfr2* heterozygosity. These studies reveal previously unrecognized *Vegfr2* gene dosage effects in tumor angiogenesis and a lack of synergy between VEGFR2 and endothelial FGFR1/2 signaling during tumor growth.

## Introduction

The Vascular Endothelial Growth Factor (VEGF) signaling pathway is essential for tumor angiogenesis^1,2^. Anti VEGF therapies clearly display anti-angiogenic efficacy in the treatment of pathological angiogenesis and some cancers. However, emerging evidence also points to significant limitations that undermine optimal clinical benefits^1,3^. Such limitations involve transient efficacy, tumor evasion, increased metastasis, and safety issues, including impaired normal vascular physiology and homeostasis^1,3,4^. The anti-VEGF therapy’s accompanying adverse effects on normal vascular physiology, for example abnormal blood pressure and thrombosis, underscores the need to understand whether target cells or gene dosage is important in tumor versus normal vascular bed homeostasis and angiogenesis. This will also establish a role for synergistic targets that can selectively block aberrant neovascular and cancer-associated angiogenesis while minimizing serious side effects. Small molecule inhibitors targeting multiple receptor tyrosine kinases (RTK) including VEGF receptors (VEGFRs) and Fibroblast Growth Factor Receptors (FGFRs) have demonstrated additive anti-tumor activity in pre-clinical models^5^. However, the relative contribution of FGFR and VEGFR pathways and compensatory mechanisms in the endothelium or other cell types is unclear.

Gene dosage effects in tumorigenesis have long been recognized in murine genetic models and humans^6–11^. However, the effects of RTK gene dosage on tumor angiogenesis have not been investigated. Although heterozygosity of *VegfA* is embryonic lethal^12^, heterozygosity of its receptor, *Vegfr2 (Flk1)* is compatible with normal vascular development and homeostasis^13,14^. Whether *Vegfr2* heterozygosity has any effect on tumorigenesis and whether other RTK pathways could be evoked to compensate for a *Vegfr2* allele loss remains unknown.

Using genetic tools in the mouse, we examine the effects of *in vivo* cell-autonomous *Vegfr2* heterozygosity, alone and in combination with endothelial *Fgfr1/2* loss, on tumor angiogenesis and growth. We demonstrate that *Vegfr2* gene dosage, but not synergy with endothelial *Fgfr1* and *Fgfr2*, is critical for tumor growth and associated tumor angiogenesis.

## Results

### *Vegfr2* heterozygosity markedly inhibits chemically-induced skin carcinogenesis and angiogenesis

**Figure 1:**
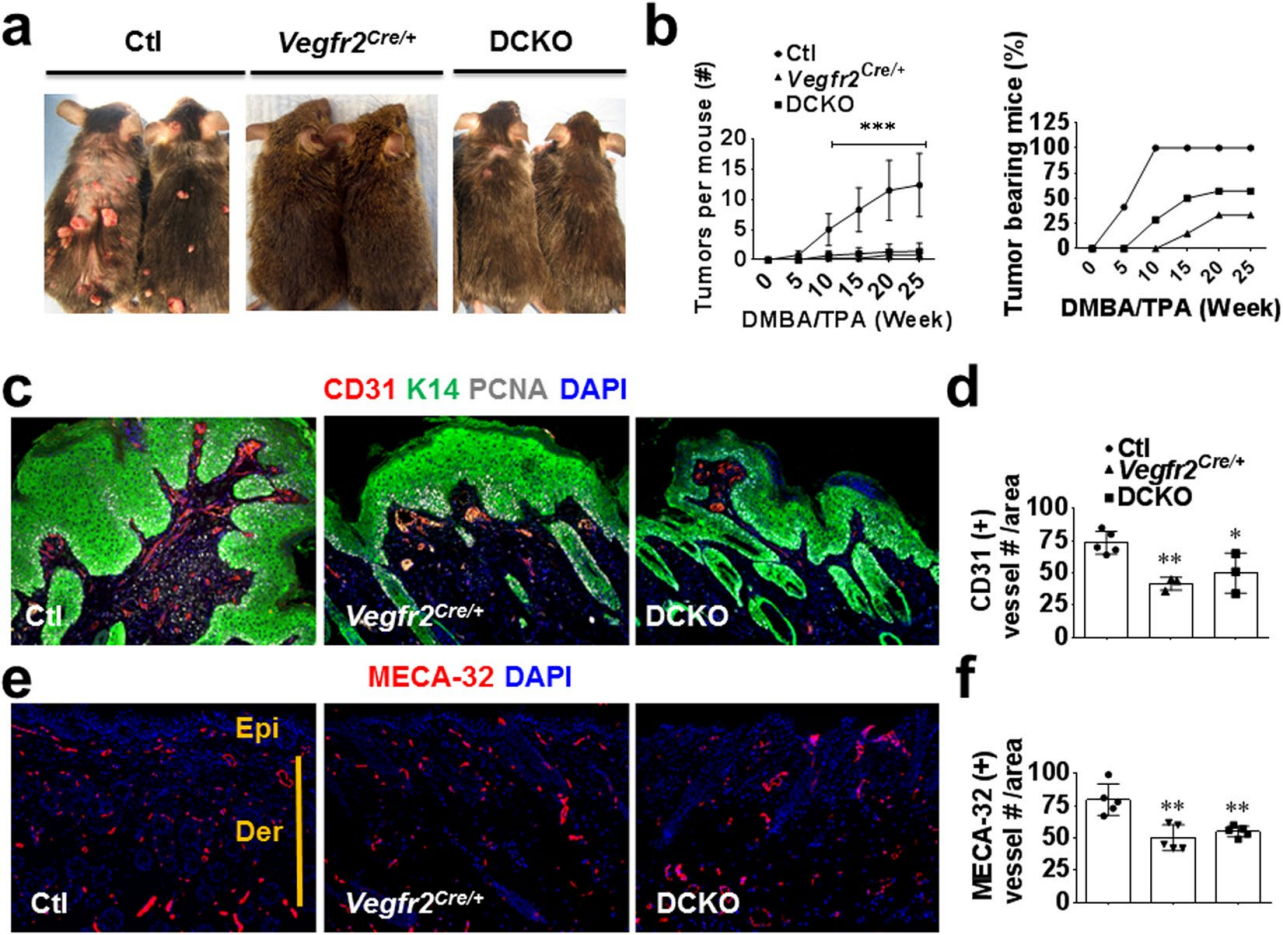
*Vegfr2* heterozygosity inhibits chemically-induced skin carcinogenesis and angiogenesis. (**a**) Increased number of papillomas (benign tumors) in control *Vegfr2*^+/+^; *Fgfr1*^*f/f*^; *Fgfr2*^*f/f*^ mice (Ctl, also referred to as DFF) compared to *Vegfr2*^*Cre*/+^ and DCKO mice. (**b**) Quantification of papilloma number and percentage of tumor occurrence in control, *Vegfr2*^*Cre*/+^, and DCKO mice (n = 15), left panel legend is applicable to the right one. (**c**) Immunofluorescence staining showing increased K14-positive papilloma cells and fewer CD31-positive endothelial cells in control versus *Vegfr2*^*Cre*/+^ or DCKO mice. (**d**) Quantification of CD31 vessels in panel C. (**e**,**f**) Immunofluorescence staining (**e**) and quantitation (**f**) showing increased Meca32- positive vessels in control versus *Vegfr2*^*Cre*/+^ or DCKO mice papilloma-bearing dorsal skin. Ctl, wild type control; Epi, epidermis; Der, dermis. Results are mean ± SEM. Mann-Whitney U test was used to analyze significance (*p < 0.05; **p < 0.01; ***p < 0.001) compared to control. Where mouse number is unstated, each symbol represents one mouse. Ear skin sections with or without papillomas were imaged with a 10X objective.

To better understand the VEGF signaling requirements in tumor angiogenesis, we assessed whether *Vegfr2*, similar to *VegfA*, exerts gene dosage effect in tumor angiogenesis. To this end, we took advantage of *Vegfr2*^*Cre*/+^ or *Vegfr2*^*LacZ*/+^ knock-in mice that are heterozygous for the *Vegfr2* gene. We also included *Fgfr1*^*f/f*^*; Fgfr2*^*f/f*^ (double floxed, DFF, used as control) and *Vegfr2*^+/+^*; Fgfr1*^*f/f*^*; Fgfr2*^*f/f*^ (double conditional knockout, DCKO) mice in the study, to determine the extent of crosstalk between VEGF and FGF signaling in tumor angiogenesis. Specifically, *Vegfr2*^*Cre*/+^; *Fgfr1*^*f/f*^*; Fgfr2*^*f/f*^ (DFF control), and *Vegfr2*^*Cre*/+^*; Fgfr1*^*f/f*^*; Fgfr2*^*f/f*^ (DCKO) mice were subjected to two-stage skin carcinogenesis^15^. Surprisingly, *Vegfr2*^*Cre*/+^ mice displayed a striking resistance to papilloma development compared to control DFF mice throughout the 25 weeks of the study as shown by a significant (p < 0.001) decrease in average number and size of papillomas per mouse (Fig. 1a,b). Interestingly, the DCKO mouse phenotype was very similar to that of *Vegfr2*^*Cre*/+^ mice, suggesting lack of synergy between VEGFR2 and endothelial FGFR1/2 signaling in this model.

To gain insight into the possible mechanisms underlying this phenotype, we determined whether there was any alteration in angiogenesis in the papilloma and adjacent skin. Immunohistological examination showed a similar reduction in vascular density in the papilloma and adjacent papilloma bearing skin in both *Vegfr2*^*Cre*/+^ and DCKO mice compared to DFF mice (Fig. 1c–f). Taken together, these data suggest a potent gene dose effect of *Vegfr2* (but not *Fgfr1/2*) on pathologic angiogenesis and tumorigenesis.

### *Vegfr2* heterozygosity impairs melanoma tumor growth and angiogenesis

**Figure 2:**
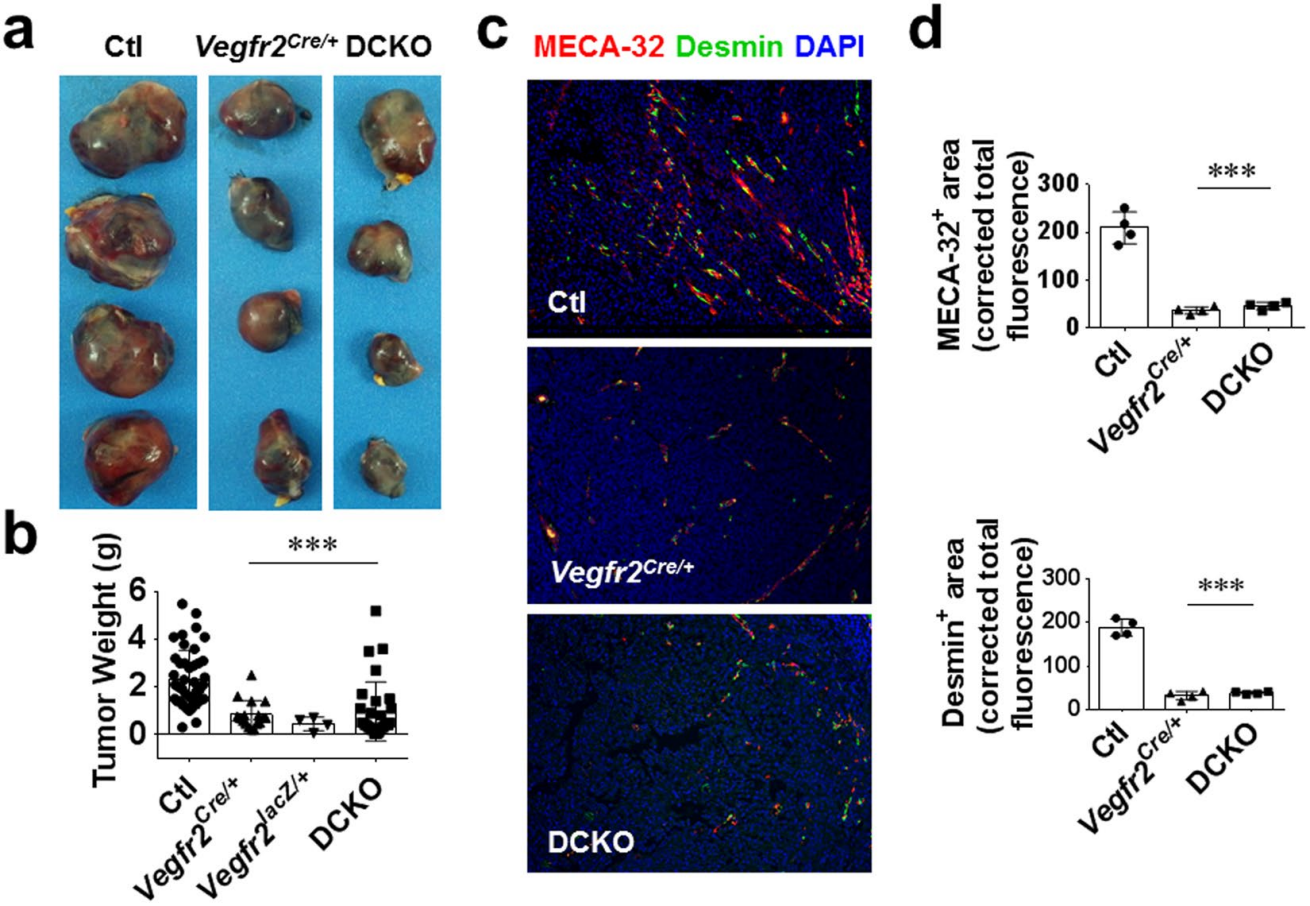
*Vegfr2* heterozygosity impairs melanoma tumor growth and angiogenesis. (**a**,**b**) B16F0 melanoma tumor weights (**a**) and quantitation (**b**) 14 days post tumor cell inoculation showing a significant decrease in *Vegfr2*^*Cre*/+^*; Vegfr2*^*lacZ*/+^ and DCKO compared to control *Vegfr2*^+/+^; *Fgfr1*^*f/f*^; *Fgfr2*^*f/f*^ (Ctl) mice. (**c**,**d**) Immunofluorescent staining (**c**) and quantitation (**d**) of Meca32 and Desmin-positive tumor vasculature showing a significant decrease in *Vegfr2*^*Cre*/+^ and DCKO compared control *Vegfr2*^+/+^; *Fgfr1*^*f/f*^; *Fgfr2*^*f/f*^ (Ctl) mice. Results are mean ± SEM. Mann-Whitney U test was used to analyze significance (***p < 0.001) compared to Ctl. Where mouse number is unstated, each symbol represents one mouse. Tumor sections (**c**) were imaged with a 10X objective.

To further study the effect of *Vegfr2* heterozygosity, alone or in combination with endothelial *Fgfr1/2* inactivation in an independent tumorigenesis model, we tested B16 melanoma cell growth when transplanted into mice. DFF, *Vegfr2*^*Cre*/+^, *Vegfr2*^*lacZ*/+^ and DCKO mice received a single subcutaneous injection of B16F0 melanoma cells in their flanks. Consistent with the results from the two-stage chemical carcinogenesis model, *Vegfr2*^*Cre*/+^*, Vegfr2*^*lacZ*/+^ as well as DCKO mice displayed a significant (p < 0.001) impairment in tumor growth compared to DFF littermate controls (Fig. 2a,b). Immunostaining and quantitation of histological sections for vascular and pericyte markers, MECA-32 and Desmin, showed a decrease in tumor angiogenesis in *Vegfr2*^*Cre*/+^ and DCKO compared to DFF control mice (Fig. 2c,d). Although, mice with floxed alleles of *Fgfrs* do not have any identified phenotypes, we nevertheless compared tumor formation in wild type (WT) mice to *Vegfr2*^+/+^; *Fgfr1*^*f/f*^; *Fgfr2*^*f/f*^ (DFF) control mice. As anticipated, these control genotypes were indistinguishable (Supplementary Fig. S1).

Analysis of VEGFR1, VEGFR2, FGFR1, FGFR3 and VE-Cadherin expression levels in B16F0 melanoma cells, and tumors isolated from control and DCKO mice showed ~30% reduced levels of VEGFR2 (but not VEGFR1) in DCKO mice compared to DFF control littermates, consistent with *Vegfr2* haploinsufficiency (Supplementary Fig. S2A,B). Additionally, VE-Cadherin and FGFR1 levels were reduced in DCKO tumors compared to control tumors, reflective of reduced tumor angiogenesis and tumor endothelial *Fgfr1* deletion (Supplementary Fig. S2A). Taken together, the B16F0 melanoma transplantation model confirmed a role for *Vegfr2* gene dosage in regulating the sensitivity to transplanted melanoma tumor growth and tumor associated neovascularization and suggests that loss of endothelial FGFR1/2 signaling does not further enhance the effect of reduced VEGFR2 expression.

### *Vegfr2* heterozygosity markedly impairs Lewis lung carcinoma (LLC) tumor growth, angiogenesis, and VEGFR2 phosphorylation

**Figure 3:**
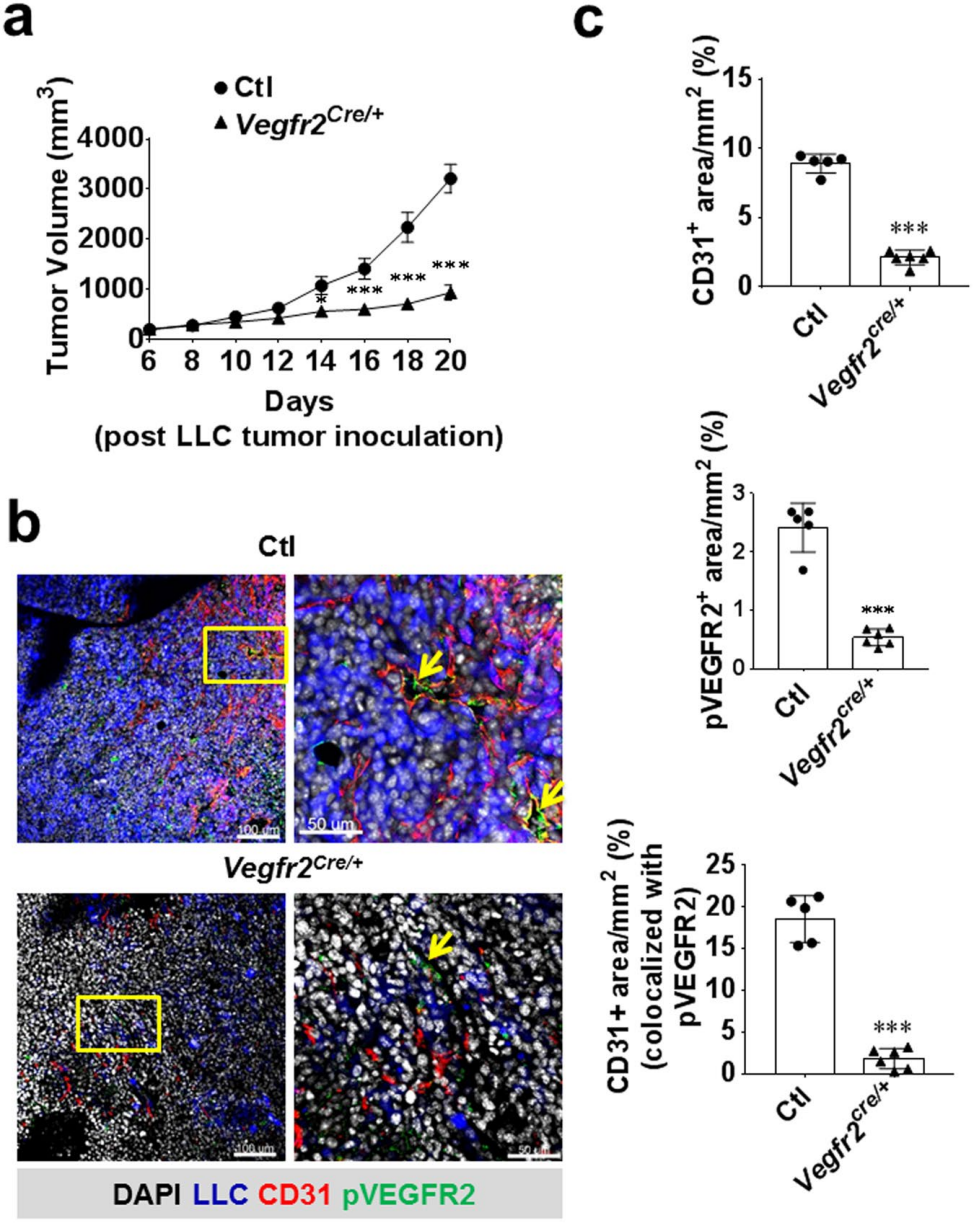
*Vegfr2* heterozygosity impairs Lewis lung carcinoma (LLM) tumor growth, angiogenesis, and VEGFR2 phosphorylation. (**a**) LLC tumor volume 20 days post tumor cell inoculation showing a significant decrease in *Vegfr2*^*Cre*/+^ compared to control wild type mice (n = 5–6). (**b**,**c**) Immunofluorescent staining (**b**) and quantitation (**c**) of CD31-positive tumor vasculature and phospho-VEGFR2 (yellow arrows) showing a significant decrease in *Vegfr2*^*Cre*/+^ compared to control mouse (n = 5–6). Two-way repeated-measures ANOVA with Sidak’s multiple comparism-test (a) and two-tailed Student’s t-test (c) were used to analyze significance (*p < 0.05; ***p < 0.001) compared to control.

We further tested the effects of *Vegfr2* heterozygosity using a third independent tumor model: subcutaneous transplantation of the Lewis Lung Carcinoma (LLC) tumor cells. As we did not observe any phenotypic difference either between *Vegfr2*^*Cre*/+^ and DCKO or DFF and wild-type (WT) in the previous two tumor models, we focused on comparing *Vegfr2*^*Cre*/+^ with littermate WT controls. Similar to our observations with the previous two models, tumor growth was markedly impaired following tumor cell injection in *Vegfr2*^*Cre*/+^ mice compared to wild type control littermates (designated Ctl) (Fig. 3a). Histological analysis revealed a significant (p < 0.001) reduction in angiogenesis similar to previous observations in the two tumor models described above (Fig. 3b,c). Furthermore, immunostaining and quantitation of histological sections for perivascular markers, Desmin and αSMA, showed a decrease in tumor perivascular coverage in *Vegfr2*^*Cre*/+^ compared to DFF control mice consistent with our observations in melanoma tumors (Supplementary Fig. S3A–D). Immunostaining, to determine whether tumor inhibition could be explained by disrupted VEGFR2 phosphorylation in LLC tumor endothelium, showed reduced VEGFR2 tyrosine phosphorylation in tumors from *Vegfr2*^*Cre*/+^ mice (Fig. 3b,c). Taken together, these data further demonstrate the dose dependency of *Vegfr2* for tumor endothelial activation, angiogenesis, and overall tumor growth.

### Hematopoietic cell *Vegfr2* deletion minimally impacts tumor growth

**Figure 4:**
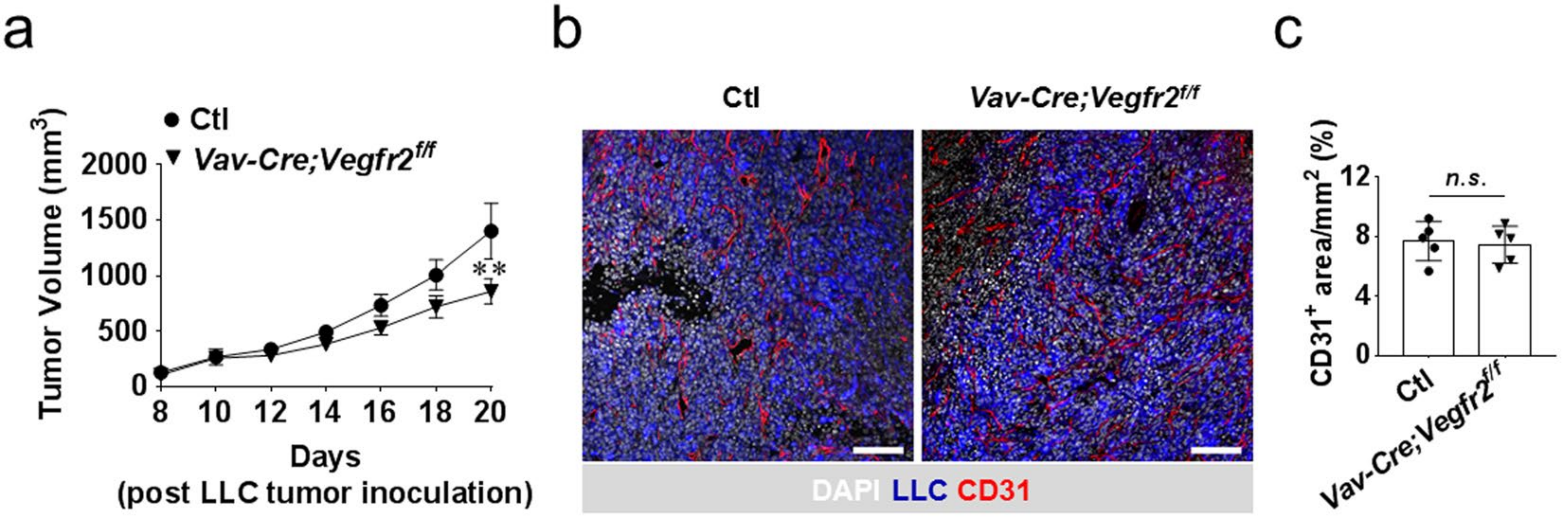
Hematopoietic cell *Vegfr2* inactivation, targeted with *Vav-Cre*, minimally impacts LLC tumor growth. (**a**) LLC tumor volume 20 days post tumor cell inoculation showing a delayed minimal decrease in tumor volume in *Vav-Cre; Vegfr2*^*f/f*^ versus *Vegfr2*^*f/f*^ control mice (designated Ctl) (n = 6). (**b**,**c**) Immunofluorescent staining (**b**) and quantitation (**C**) of CD31-positive tumor vasculature showing no impairment in tumor angiogenesis *Vav-Cre; Vegfr2*^*f/f*^ versus control mouse (n = 5). Two-way repeated-measures ANOVA with Sidak’s multiple comparism-test (a) and two-tailed Student’s t-test (c) were used to analyze significance (**p < 0.01) compared to control. ns, not significant. Scale bar, 100 μm.

Defects in tumor angiogenesis in *Vegfr2*^*Cre*/+^ mice suggested the requirement of endothelial *Vegfr2* in tumor angiogenesis. Given that *Vegfr2*^*Cre*/+^ targets also hematopoietic cells^16^, we aimed at ruling out a possible contribution of hematopoietic *Vegfr2* in tumor angiogenesis by specifically inactivating *Vegfr2* in hematopoietic cells. The, *Vav-Cre* allele, which preferentially targets hematopoietic cells^17^ was used to delete *Vegfr2*. Mice with the genotype *Vav-Cre; Vegfr2*^*f/f*^ were subcutaneously transplanted with LLC tumor cells and monitored for tumor growth for 20 days. Compared to *Vegfr2*^*Cre*/+^ mice, a minimal tumor growth inhibition was observed in *Vav-Cre; Vegfr2*^*f/f*^ mice (Fig. 4a). Notably, *Vav-Cre; Vegfr2*^*f/f*^ mice did not show any impairment in tumor angiogenesis, suggesting that the small reduction in tumor growth is endothelial cell independent (Fig. 4b). We confirmed efficient deletion of *Vegfr2* in whole blood or CD31^−^ CD45^+^ hematopoietic cells from the lung, but not in CD31^+^ CD45^−^ endothelial cells (Supplementary Fig. S4). Taken together, these data suggest a minimal role for hematopoietic *Vegfr2* during tumor growth.

### Lymphocyte or macrophage infiltration into the skin in response to TPA was unaffected by the *Vegfr2* and endothelial *Fgfr1/2* deficiency

**Figure 5:**
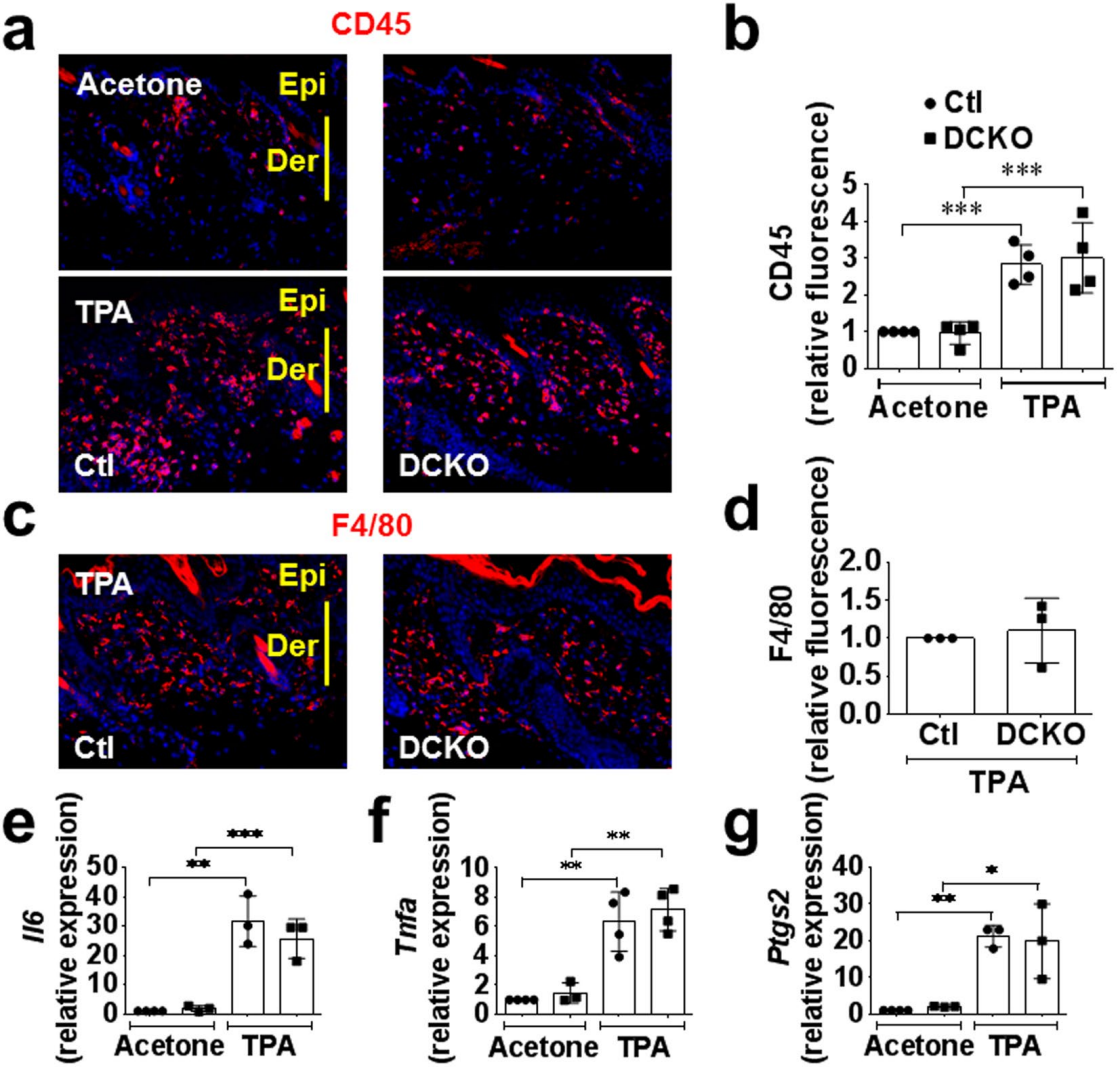
Lymphocyte or macrophage infiltration into the skin in response to TPA was unaffected by *Vegfr2* and endothelial *Fgfr1/2* deficiency. (**a**,**b**) Immunofluorescence staining (**a**) and quantitation (**b**) showing increased CD45-positive inflammatory cells seven days post TPA challenge compared to vehicle (acetone). No difference was observed in both DFF control (Ctl) and DCKO responses. (**c**,**d**) Immunofluorescence staining (**c**) and quantitation (**d**) showing increased F4/80-macrophages seven days post TPA challenge. No difference was observed in control and DCKO mice. (**e**,**f**) Increased inflammatory cells markers, *Tnfa*, *Il6* and *Ptgs2*, seven days post TPA challenge in both control and DCKO mice compared to vehicle treated mice. No difference was observed between genotypes. For quantitative data, each symbol represents one mouse. Mann-Whitney U test was used to analyze significance (*p < 0.05; **p < 0.01; ***p < 0.001) compared to vehicle (Acetone). Ear skin (**a**,**c**) sections were imaged with a 10X objective.

Inflammatory cells are known to support tumor angiogenesis. To exclude a possible role for *Vegfr2* heterozygosity in immune cell infiltration, we profiled the expression of key inflammatory markers in response to TPA challenge at the same concentration used to promote tumorigenesis in the DMBA-induced chemical carcinogenesis model. Histologic sections from DFF (*Vegfr2*^+/+^; *Fgfr1*^*f/f*^; *Fgfr2*^*f/f*^) control and DCKO (*Vegfr2*^*Cre*/+^; *Fgfr1*^*f/f*^; *Fgfr2*^*f/f*^) mouse skin was stained for CD45 and F4/80 to identify leucocytes and macrophages, respectively. In response to twice weekly TPA treatments, there was a 3-fold increase in the number of CD45 positive inflammatory cells in both control and DCKO skin (Fig. 5a,b). However, there was no difference in CD45^+^ hematopoietic cell infiltration between the two genotypes. Also, control and DCKO mice showed similar expression of F4/80 positive macrophages after three TPA treatments in a seven-day study (Fig. 5c,d). Consistent with these data, expression levels of inflammatory chemokines such as *Tnfa*, *Il6*, and *Ptgs2* (*Cox2*) were increased (6–15 fold) in response to TPA treatment, but showed no difference relative to genotype (Fig. 5e–g). It is thought that VEGFR1 provides a migratory signal to inflammatory cells. We therefore examined *Vegfr1* and *Vegfr2* mRNA levels in skin challenged with TPA for seven days. While, *Vegfr1* RNA levels were increased by four-fold in response to TPA, there was no difference between control and DCKO mice (Supplementary Fig. S5A). TPA also induced an increase in *Vegfr2* mRNA levels in both control and DCKO mice; however, the increase in *Vegfr2* RNA levels in DCKO mice was less than that in control mice (Supplementary Fig. S5B). Together, these data show that lymphocyte or macrophage infiltration into the skin in response to TPA was not affected by *Vegfr2* and endothelial *Fgfr1/2* deficiency.

## Discussion

The VEGF signaling pathway is the primary angiogenic target and is successfully being used in the clinic for the treatment of various malignancies by blocking either the ligand, VEGFA, or the receptor, VEGFR2^18–20^. However, while cancer patients clearly benefit from anti-VEGF therapies, it comes with the cost of vascular-related side effects and complications, including abnormal blood pressure and thrombosis^3^. Additionally, the benefit of anti-VEGF therapy is short-lived and tumors inevitably escape this treatment and continue to grow^21–23^. Of the known anti-VEGF mechanisms of evasion, induction of compensatory pathways including FGFR activation is well documented^23^.

In the current study, we found that VEGFR2 signaling in hematopoietic cells was not required for tumor angiogenesis, as *Vegfr2* deletion in hematopoietic cells minimally affected tumor angiogenesis. Interestingly, a recent study reported that VEGFR2 was required in hematopoietic cells for glioma progression; however, tumor angiogenesis was not examined in this study^24^. Future studies of contribution of VEGF signaling in hematopoietic cells to tumor growth independent of angiogenesis are therefore warranted.

While tumor growth and tumor angiogenesis was strikingly impaired by *Vegfr2* heterozygosity, simultaneous inactivation of endothelial *Fgfr1/2* did not enhance the tumor inhibitory effects, despite studies showing additive efficacy using pharmacological small molecular inhibitors targeting multiple tyrosine kinases^5,25^. However, since endothelial cells also express *Fgfr3* (but not *Fgfr4*)^26^, and upregulate *Fgfr3* expression in the absence of *Fgfr1*^27^, it is possible that *Fgfr3* could compensate for the *Fgfr1* and *Fgfr2* deficiency in tumor endothelial cells. Potential contributions of FGFR3 should be further investigated. Alternatively, non-endothelial FGFR signaling may contribute to cancer growth and choosing drugs with moderate VEGFR2 and FGFR1/2 inhibitory properties still should be considered and may be more efficacious and less toxic than strongly inhibiting both pathways. The abundant expression of FGFR1 in melanoma tumor cells shown here (Supplementary Fig. SA) supports a potential non-endothelial role for FGFR1 in the growth of this tumor. In addition, the context dependence of FGF signaling in different cell types in tumors may explain differences in study outcomes between this work and others^28^. Future studies will be required to test the ability of mice conditionally lacking FGFRs 1, 2, and 3 in endothelial cells to support tumor growth with and without a wild type *Vegfr2* allele. Lastly, while tumor angiogenesis clearly plays a role in the tumor phenotypes described in our studies, potential contributions of angiogenesis independent mechanisms such as vascular co-option, a process involving crawling along existing host vasculature by cancer cells to gain access to blood supply^29^, cannot be ruled out, particularly regarding the consequence of endothelial FGFR1/2 deletion.

Of note, findings in the current study showing a lack of synergy between VEGFR2 and FGFR1/2 signaling during tumor growth are distinct from our previous study where *Vegfr2* heterozygosity showed no effect on injury response, while *Fgfr1/2* inactivation in endothelial and hematopoietic cells was required for neoangiogenesis in response to injury^13^. Additionally, endothelial inactivation of *Fgfr1/2* impaired the heart’s response to ischemia-reperfusion injury^30^. The role of *Vegfr2* haploinsufficiency was not investigated in this model. These differences between FGFR and VEGFR2 signaling requirements highlight speculated and increasingly appreciated differences between the angiogenic response and signaling requirements of different vascular beds in the context of injury response and cancer. Future studies testing the role of *Vegfr2* heterozygosity in other non-cutaneous tumor models and exploring whether there is a different outcome using inducible systems to temporally inactivate one allele of *Vegfr2* are warranted.

**Figure 6:**
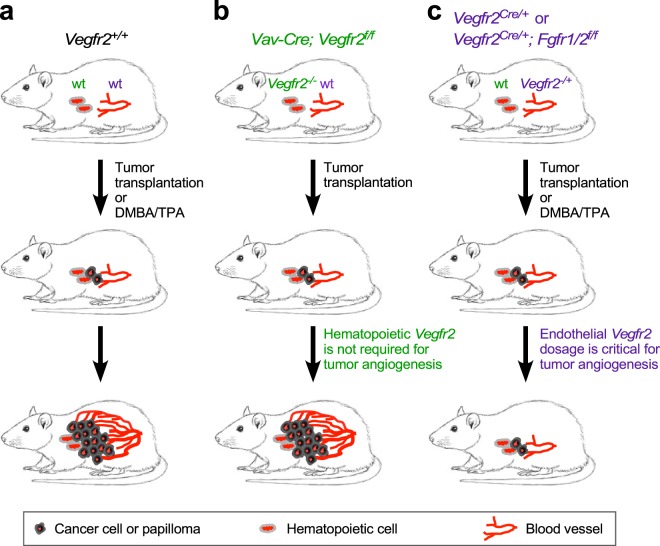
A model depicting *Vegfr2* heterozygosity effect on tumor growth and accompanying angiogenesis. (**a**) Mice containing wild type (wt) alleles of *Vegfr2* and *Fgfr1/2* develop tumors in a skin carcinogenesis or transplantation model. (**b**) Loss of *Vegfr2* in hematopoietic cells does not affect tumorigenesis. (**c**) *Vegfr2* heterozygosity (in endothelial cells and hematopoietic cells) impairs tumor growth and angiogenesis. Additional loss of endothelial *Fgfr1* and *Fgfr2* does not further impair tumor growth or angiogenesis in *Vegfr2* heterozygous mice.

The data presented here suggests that while *Vegfr2* heterozygosity strikingly leads to defects in tumor growth and tumor angiogenesis in all the three tumor models, additional loss of endothelial *Fgfr1* and *Fgfr2* displays no further effects on tumor growth and tumor angiogenesis. The gene dosage of *Vegfr2*, but not endothelial *Fgfr1/2*, in tumor growth and pathologic neovascularization (Fig. 6) suggests that for optimal anti-angiogenic response and to minimize toxicity to the normal vasculature, partial pharmacological blockade of VEGFR2 signaling may be more efficacious and with fewer side effects than complete inhibition of VEGFR2 in some tumor types.

## Methods

### Mice

*Vegfr2*^*Cre*/+^^14^, *Vegfr2*^*LacZ*/+^^31^, *Vegfr2*^*Cre*/+^; *Fgfr1*^*f/f*^; *Fgfr2*^*f/f*^ (DCKO mice)^13^*, Vegfr2*^*f/f*^^32^, and *Vav-Cre*^17^ mice were previously described. DCKO mice were shown to have an 84% reduction in *Fgfr1* and 87% reduction in *Fgfr2* expression in sorted lung endothelial cells and were phenotypically normal as adults^13^. *Vegfr2*^*LacZ*/+^ or *Vegfr2*^*Cre*/+^ (*Vegfr2*^+/−^), *Vegfr2*^+/+^; *Fgfr1*^*f/f*^; *Fgfr2*^*f/f*^ (double floxed, DFF, control, designated Ctl), and *Vegfr2*^*Cre*/+^*; Fgfr1*^*f/f*^*; Fgfr2*^*f/f*^ (double conditional knockout, DCKO) mice were maintained on an inbred C57BL/6 genetic background following ten generations of backcrossing to wild type C57BL/6 J mice. *Vav-Cre* and *Vegfr2*^*f/f*^ mice were also maintained on a C57BL/6 genetic background.

### Cell lines

B16 melanoma cells (provided by Kathy Weilbaecher, Washington University) and Lewis lung carcinoma (LLC) cells (obtained from Alexander Krupnick, Washington University) were maintained in High Glucose DMEM (ThermoFisher Inc.), supplemented with 10% (v/v) FBS, 100 unit/ml penicillin and 100 μg/ml streptomycin.

### Cancer models

For two stage chemical carcinogenesis, the back skin of each age-matched mouse was treated once with DMBA and repetitively with TPA for 25 weeks as previously described^15,33,34^. Specifically, the back skin of each age-matched (8–10 weeks old) mouse was shaved under anesthesia two days before topical treatment with a single dose of DMBA (0.1 mg/ml in acetone [or 25 µg in 200 µl acetone], MilliporeSigma Inc.). Beginning one week later, mice were topically treated twice weekly with TPA (12.5 µg in 200 ml acetone per treatment; MilliporeSigma Inc.) and monitored for 25 weeks. Control mice (n = 4) of each genotype were treated with acetone only. Papillomas >1 mm in diameter were counted over the course of TPA challenge and at necropsy.

For the B16 melanoma cell model^35,36^ 1 × 10^6^ cells were injected subcutaneously in the hind limb flank under anesthesia. Tumor growth was monitored and recorded over the course of the study. Tumors were excised and tumor weight determined at necropsy on day 14.

For the LLC cell model, 1 ml of growth factor reduced Matrigel: Matrix protein HC (Cat: 354248; Corning) was mixed with 1 ml of tumor cell suspension (2 × 10^6^/ml in PBS); 100 µl of tumor cell-Matrigel mixture was injected subcutaneously to the back of mice. Tumor growth was monitored and tumor volume recorded using digital slide calipers (VWR International) every other day from day 6 to day 20 after tumor cell inoculation. Tumor volume was calculated by the equation, Volume = (largest diameter) × (smallest diameter)^2^ × 0.5.

### TPA-induced cutaneous inflammation

The dorsal back (shaved under anesthesia) or external ear skin was challenged with TPA (12.5 µg in 200 µl Acetone) or vehicle (acetone) twice per week. Mice were sacrificed on day 7, skin tissues were collected and processed for immunostaining or gene expression analysis.

### Tissue harvest, Immunostaining, and Microscopy

Tissues were isolated and immunostained as previously described^13,37^. At necropsy, papilloma, or papilloma bearing skin, B16F0 melanoma tumors, or LLC carcinoma tumors were excised, fixed either in 10% (vol/vol) buffered formalin for frozen sectioning or 70% (vol/vol) cold ethanol for paraffin embedding. Tumors harvested in 10% buffered formalin were immersed in 30% (w/v) sucrose solution for 48 h to cryo-protect the tissue, frozen in NEG-50 frozen section medium (Cat: 6502, ThermoFisher scientific) using liquid nitrogen and 2-methylbutane, and sectioned (16 µm) using a Cryostat Cryocut Microtome (Leica, CM1850, Nussloch, Germany). Frozen sections were first blocked at room temperature using freshly made blocking buffer (3% essentially IgG free BSA (Cat: A9085, MilliporeSigma Inc.), 0.3% Triton X-100, and Fc blocker (Cat: 101301, Biolegend)) to prevent non-specific binding. Additionally, formalin fixed paraffinized tissue sections (5 µm) were deparaffinized, washed in PBS, underwent antigen retrieval using a pressure cooker and citrate buffer (pH6) and blocked with antibody diluent plus 10% goat serum for 30 min at room temperature. Immunostaining of both the frozen and paraffinized tissue sections was carried out using the following reagents. For primary antibodies (incubation at 4 °C overnight except otherwise stated): Meca32 (1:50, 550563; BD Bioscience), rat anti-mouse CD31, clone SZ31 (1:50, DIA-310; Dianova), hamster anti-mouse CD31 (1:400) (Cat: MA3105, ThermoFisher Scientific), rabbit anti-mouse K14 (1:2,000, PRB-155P; Covance), anti-mouse proliferating cell nuclear antigen (PCNA) (1:20, sc-56; Santa Cruz Biotechnology), F4/80 (1:500, MCA497R; Serotec), rat anti-mouse: CD45 (1:300; 550539; BD Bioscience), Phospho-VEGF Receptor-2 (Tyr1175)(19A10) Rabbit mAb (1:1000; Cat: 2478, Cell Signaling Technology), and rabbit anti-mouse Phospho-VEGF Receptor-2 (Y951) (1:200) (Cat: 4991 S, Cell Signaling Technology). For secondary antibodies: Alexa Fluor 594-labelled goat anti-rat (1:5500, A11007: Molecular probes), Alexa Fluor 568 labeled goat anti-hamster (1:500) (Cat: A-21112, ThermoFisher), Alexa Fluor 620-labbled goat anti-mouse (1:500, A21235; Molecular Probes, Alexa Fluor 488-labelled goat anti-rat (1:500, A11006; Molecular Probes), and Alexa Fluor 647 labeled goat anti-rabbit (1:500) (Cat: A-21245, ThermoFisher Scientific) incubate for one hour at room temperature. Tissue sections for fluorescence microscopic imaging were mounted with SlowFade Gold anti-fade reagent with DAPI (S36938; Invitrogen, Molecular Probes) or ProLong Diamond Antifade mountant (Cat: P36970, ThermoFisher scientific) and imaged on a Zeiss ApoTome with 10X and 20X objectives.

### Vascular Density Quantification

Vascular density was assessed by counting the number of CD31- or Meca32-positive vessels in three fields (10X objective) per section per mouse (n = 3–16 mice). Alternatively, at least three images from every section per mouse (n = 5 mice) were processed using ImageJ software (NIH) to quantify CD31 and pFLK1 positive areas.

### Immunoblotting

Western blots were performed as previously described^37^. Briefly, tissues were lysed in RIPA buffer with freshly added protease and phosphatase inhibitors (P8340, P5726 AND P0044; MilliporeSigma Inc.). Equal amounts of protein lysates were separated on SDS 4–12% polyacrylamide Tris-glycine gradient Midi gels (WT4121BX10; Invitrogen) and transferred to PVDF membranes (iBlot Gel Transfer Stacks PVDF Regular, IB4010-01; Invitrogen). Immunobloting was performed as previously described^13^. Briefly, skin, papilloma and tumors were excised. Tissues were homogenized in RIPA buffer with freshly added protease and phosphate Inhibitor cocktail and centrifuged to obtain lysates. Protein quantification was performed on clarified tissue lysates and then subjected to SDS-PAGE. Membranes were probed with the following primary antibody at 4 °C overnight except as otherwise stated: rabbit monoclonal VEGFR1 (ab3215, Abcam), rabbit monoclonal VEGFR2 (2479, Cell Signaling), VE-Cadherin (sc-6458; Santa Cruz), rabbit polyclonal FGFR1 (sc-121; Santa Cruz, FGFR3 antibody (generated by David M. Ornitz laboratory at Washington University in St. Louis). After overnight incubation, membranes were washed 3x and incubated for 1 hour at room temperature in horseradish peroxidase-conjugated anti-rabbit (1:2000, sc-2301; Santa Cruz) or anti-goat IgG antibodies (sc-2304; Santa Cruz in TBST with 5% nonfat milk and developed in SuperSignal West Femto Maximum Sensitivity Substrate (34096; Thermo Scientific). Membranes were stripped in multiple washes of boiled 100 mM glycine and washed three times in TBST for 10 min each (per iBlot stripping protocol; Invitrogen) at room temperature. Stripped membranes were then reprobed for β-tubulin (1:50,000, ab6046; Abcam). Protein bands were imaged using a BioRad ChemiDoc MP Imager.

### Gene Expression Analysis

Tissue samples were homogenized in 1 ml TRIzol (#15596-018; Invitrogen). Total RNA was isolated according to manufacturer’s instructions. Total RNA, following TRIzol extraction, was further purified using RNeasy kit (Cat: 74106; Qiagen) and DNAse-treated on a column with RNAse Free DNAse set (Cat: 79254; Qiagen). Total RNA was transcribed into cDNA as per manufacturer’s protocol. Real-time RT-PCR analysis was performed using inventoried TaqMan^®^ gene expression primers and probes (Additional file 1: Table S1). Target cDNAs were normalized to *Gapdh*. Quantitative RT-PCR assays were run using a TaqMan^®^ Applied Fast Advanced Mastermix according to the manufacturer’s protocols (Cat: 4444557; ThermoFisher).

### Statistical Analysis

Data are reported as mean ± SD and changes with P values less than 0.05 were considered statistically significant. Data were analyzed using unpaired Student’s t-test, one-way ANOVA, and two-way repeated-measures ANOVA (Prism 7). Numbers of mice used per group per experiments are stated in the figure legends.

### Study Approval

Mice were housed in a pathogen-free facility and handled in accordance with standard use protocols, animal welfare regulations, and the NIH Guide for the Care and Use of Laboratory Animals. All protocols were approved by the Washington University in St. Louis Animal Care Committee and Institutional Biosafety.

## Electronic supplementary material

Supplemental Information
