# Supplementary material for: Impaired tumor growth and angiogenesis in mice heterozygous for Vegfr2 (Flk1)
Source: Sci Rep. 2018 Oct 3;8:14724. doi: 10.1038/s41598-018-33037-2 (PMC6170482; doi:10.1038/s41598-018-33037-2)
Supplement: Supplementary file 1 — Supplemental Information [file 41598_2018_33037_MOESM1_ESM.pdf]

Impaired tumor growth and angiogenesis in mice heterozygous for *Vegfr2 (Flk1)*

Sunday S. Oladipupo[ac,*], Ashraf Ul Kabir[b], Craig Smith[a], Kyunghee Choi[bd], and David M. Ornitz[a,*]

Departments of [a]Developmental Biology and [b]Pathology and Immunology, Washington University School of Medicine, St. Louis, MO 63110.

[c]Present Address: Bio-TDR, Lilly Research Laboratories, Eli Lilly and Company, Indianapolis, IN 46285.

[d]Graduate School of Biotechnology, Kyung Hee University, Yong In, South Korea.

*Correspondence: oladipupo_sunday_s@lilly.com or dornitz@wustl.edu.

Supplementary Fig. S1: Target genes and respective Taqman probe product numbers

**Gene**	TaqMan® probe catalog #
Tnfa	Mm00443260_g1
Il-6	Mm00446190_m1
Ptgs2	Mm00478374_m1
Vegfr1	Mm00438980_m1
Vegfr2	Mm01222421_m1
Gapdh	Mm99999915_g1

SUPPLEMENTARY FIGURE LEGENDS

Supplementary Fig. S1. *Vegfr2* heterozygosity impairs melanoma tumor growth. B16F0 melanoma tumor 14 days post tumor cell inoculation showing a marked decrease in tumor weight in $Vegfr2^{Cre/+}$, $Vegfr2^{lacZ/+}$, and DCKO mice compared to controls wild type (WT), $Vegfr2^{+/+}$; $Fgfr1^{f/f}$; $Fgfr2^{f/f}$ (DFF) mice. Results are mean ± SEM. Mann-Whitney U test was used to analyze significance (*** $p<0.001$) compared to control. Where mouse number is unstated, each symbol represents one mouse.

Supplementary Fig. S2. VEGFR2 and FGFRs protein levels in melanoma cells and tumors grown in control and DCKO mice. (a) Western blot analysis of B16F0 melanoma cells before and after injection into control and DCKO mice displayed efficient *Fgfr1* deletion as shown by minimal FGFR1 protein detection in tumors from DCKO mice compared to control mice and B16F0 cells. FGFR3 and VEGFR1 levels were similar in tumors from control and DCKO mice and FGFR3 was highly expressed in B16F0 cells. VEGFR2 was not detected in B16F0 melanoma cells and was reduced in tumors from DCKO mice (a, b), consistent with *Vegfr2* heterozygosity in these mice. VE-Cadherin levels were reduced in DCKO tumors compared to controls. Cropped gels are displayed.

Supplementary Fig. S3. *Vegfr2* heterozygosity impairs Lewis lung carcinoma (LLM) tumor neovascularization. (a, c) Immunofluorescence staining of LLC tumors 20 days post implantation showing a marked decrease in Desmin (a) or αSMA (c) in CD31+ areas in $Vegfr2^{Cre/+}$ compared to control wild type mice (n=3). (b, d) Quantitation of immunofluorescence staining in a, and c, showing $Desmin^{+}$, $CD31^{+}$ area (b) and

αSMA$^+$, CD31$^+$ area (d). Two-tailed Student's t-test was used to analyze significance (* $p<0.05$, ** $p<0.01$) compared to control. Scale bars, 100 μm (left panels: a,c) or 4.0 μm (right panels: a,c).

Supplementary Fig. S4. *Vegfr2* is efficiently deleted in hematopoietic cells in *Vav-Cre; Vegfr2*$^{f/f}$ mice. Genomic DNA PCR analysis of peripheral blood (a), CD31$^-$CD45$^+$ sorted hematopoietic cells from lungs (b), and CD31$^+$CD45$^-$ sorted endothelial cells from lungs (c) from 2 independent *Vav-Cre*; *Vegfr2*$^{f/f}$ and control (*Vegfr2*$^{f/f}$) mice are shown. Note that a faint deleted *Vegfr2* band is observed in the endothelial cells of the *Vav-Cre*; *Vegfr2*$^{f/f}$ mice, which reflects that *Vav-Cre* targets a small number of endothelial cells during development. Cropped gels are displayed.

Supplementary Fig. S5. *Vegfr1* and *Vegfr2* mRNA expression in control and DCKO mouse skin seven days post TPA challenge. (a) *Vegfr1* mRNA levels were similarly induced in the skin of both control (Ctl) and DCKO mice. (b) *Vegfr2* mRNA levels were induced in the skin of both control and DCKO mice, but levels were lower in DCKO mice compared to induced control mice, consistent with *Vegfr2* heterozygosity. Results are mean ± SEM. Mann-Whitney U test was used to analyze significance (** $p= 0.006$; *** $p=0.0002$; $^{\#}$ $p=0.038$; $^{\#\#\#}$ $p<0.0001$) compared to control.

SI Figure 1

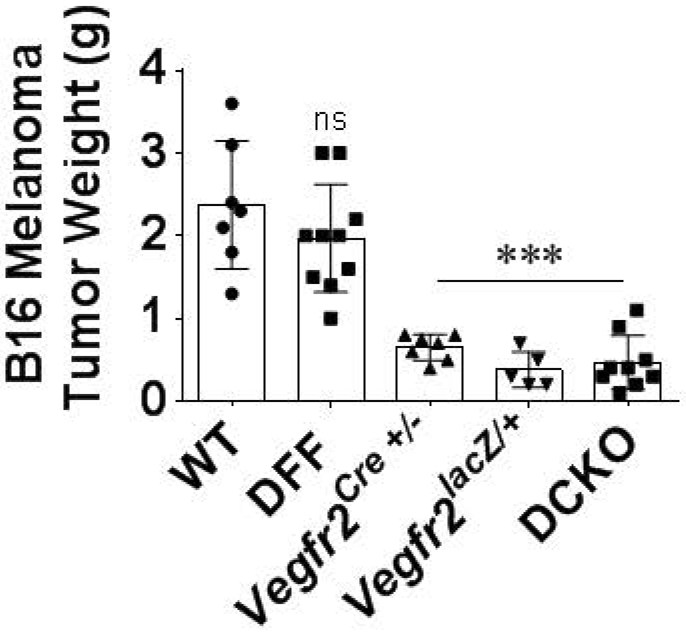
B16 Melanoma
Tumor Weight (g)
4
3
2
1
0
ns
***
WT
DFF
Vegfr2Cre +/-
Vegfr2lacZ/+
DCKO

SI Figure 2

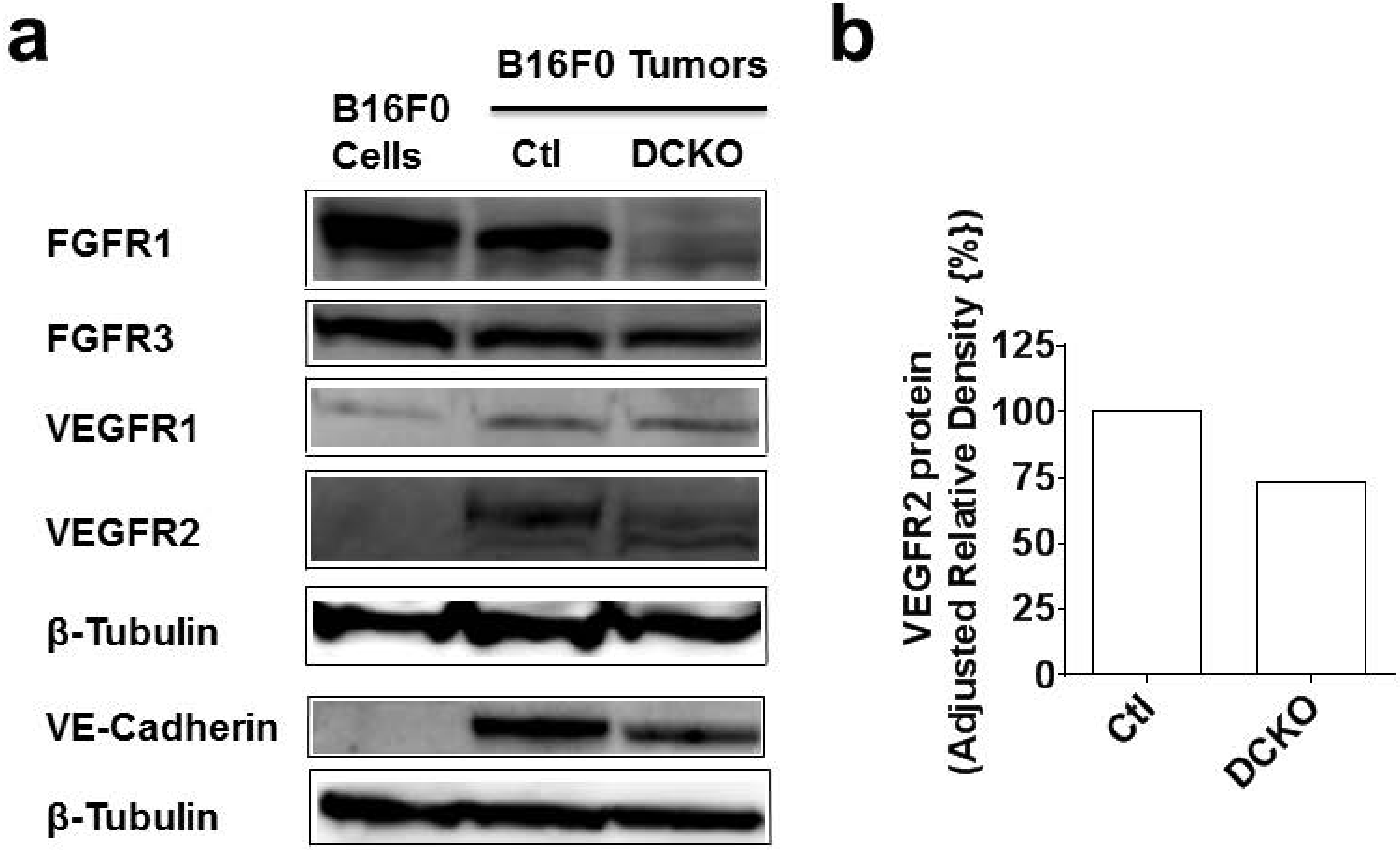

a
B16F0 Tumors
B16F0 Cells
Ctl
DCKO
FGFR1
FGFR3
VEGFR1
VEGFR2
β-Tubulin
VE-Cadherin
β-Tubulin
b
VEGFR2 protein (Adjusted Relative Density {%})
125
100
75
50
25
0
Ctl
DCKO

SI Figure 3

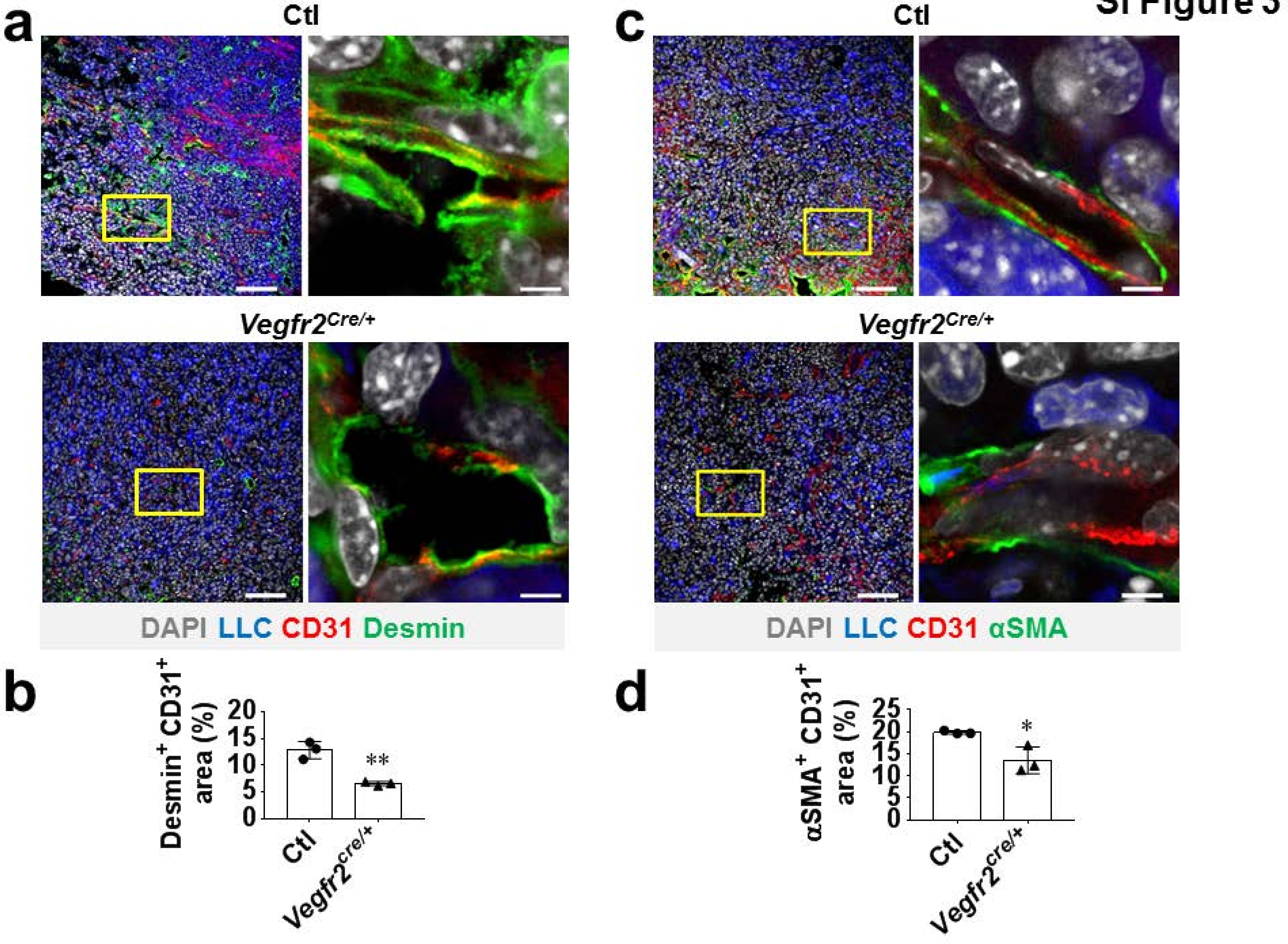
a
Ctl
Vegfr2$^{Cre/+}$
DAPI LLC CD31 Desmin
c
Ctl
Vegfr2$^{Cre/+}$
DAPI LLC CD31 αSMA
b
Desmin^{+} CD31^{+} area (%)
20
15
10
5
0
**
Ctl
Vegfr2$^{cre/+}$
d
αSMA^{+} CD31^{+} area (%)
25
20
15
10
5
0
*
Ctl
Vegfr2$^{cre/+}$

SI Figure 4

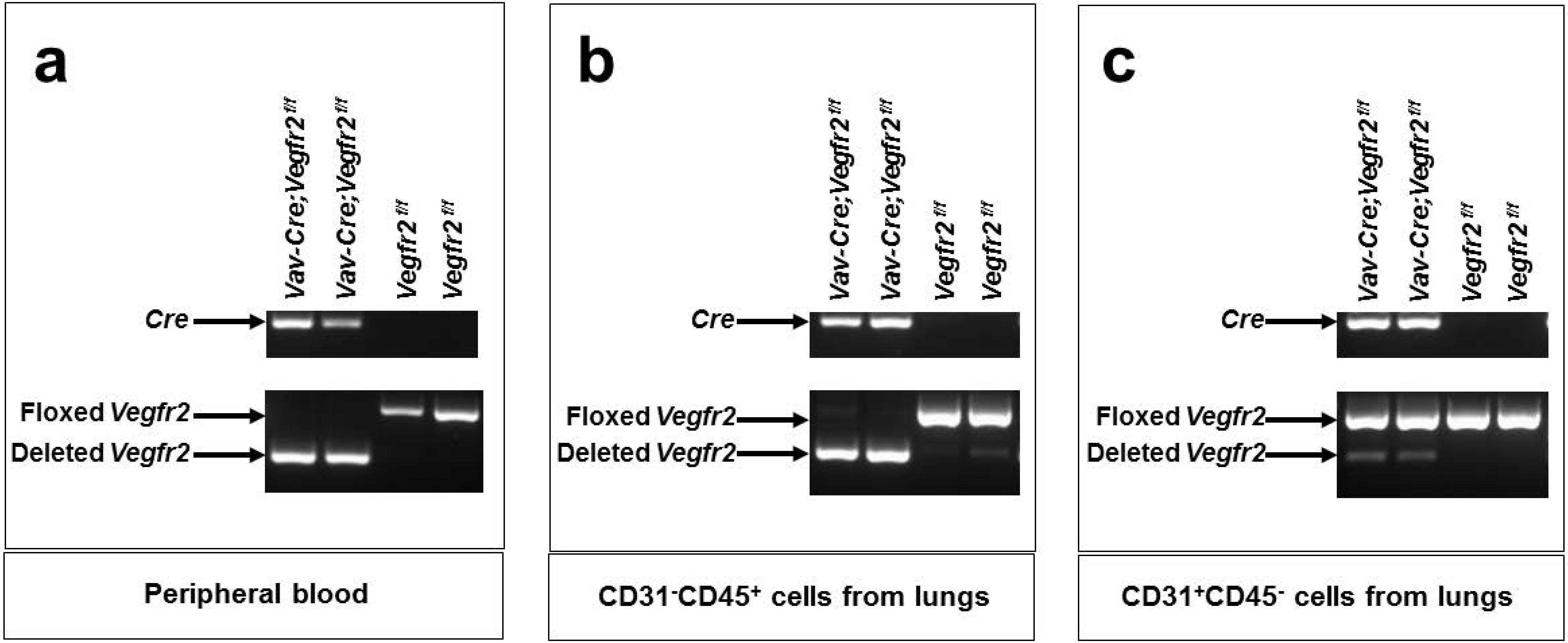
a
Vav-Cre;Vegfr2 f/f
Vav-Cre;Vegfr2 f/f
Vegfr2 f/f
Vegfr2 f/f
Cre
Floxed Vegfr2
Deleted Vegfr2
Peripheral blood
b
Vav-Cre;Vegfr2 f/f
Vav-Cre;Vegfr2 f/f
Vegfr2 f/f
Vegfr2 f/f
Cre
Floxed Vegfr2
Deleted Vegfr2
CD31-CD45+ cells from lungs
c
Vav-Cre;Vegfr2 f/f
Vav-Cre;Vegfr2 f/f
Vegfr2 f/f
Vegfr2 f/f
Cre
Floxed Vegfr2
Deleted Vegfr2
CD31+CD45- cells from lungs

SI Figure 5

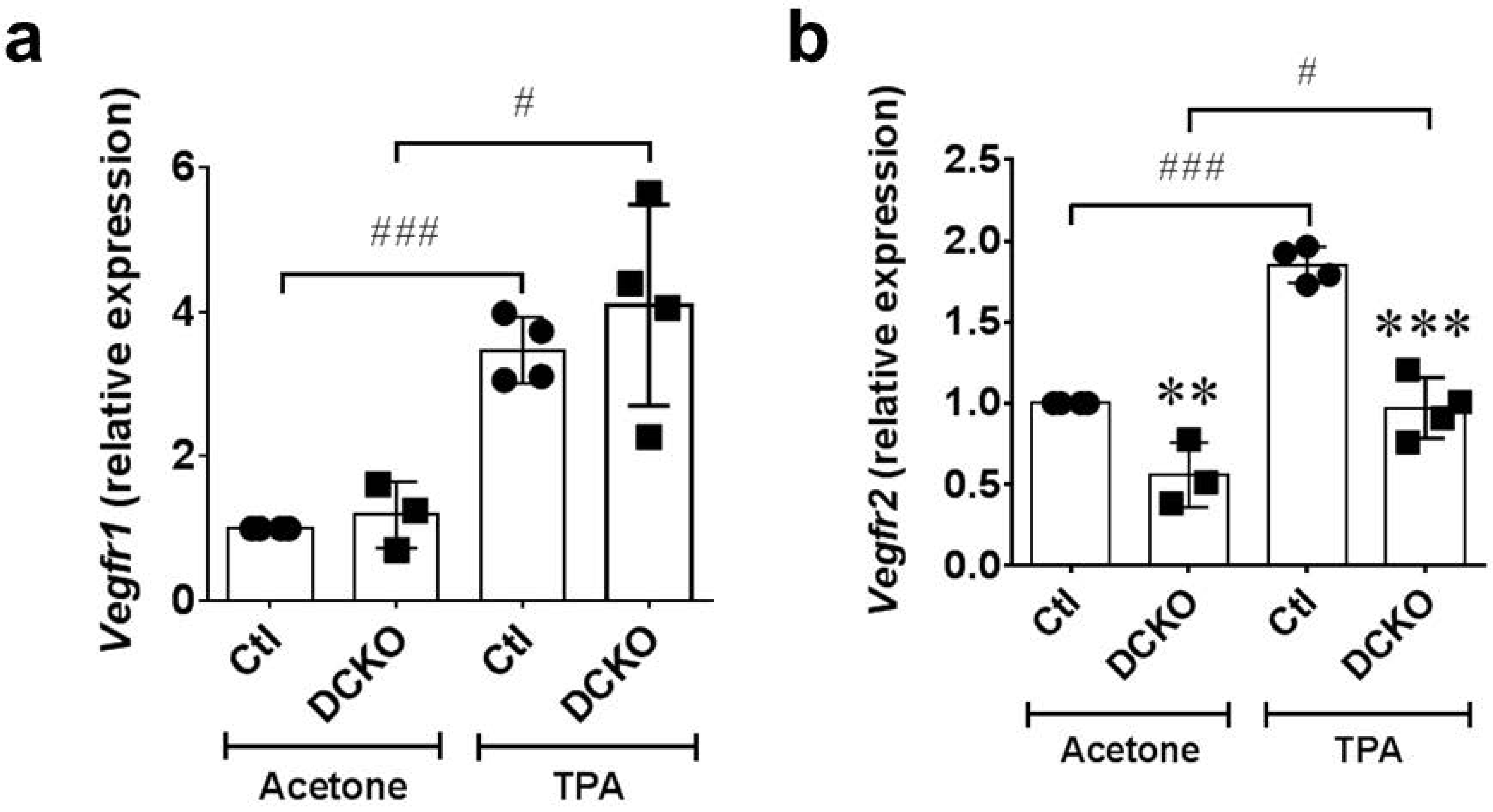
a
b
Vegfr1 (relative expression)
0
2
4
6
###
#
Ctl
DCKO
Ctl
DCKO
Acetone
TPA
Vegfr2 (relative expression)
0.0
0.5
1.0
1.5
2.0
2.5
###
#
**
***
Ctl
DCKO
Ctl
DCKO
Acetone
TPA
